# Spontaneous renal tract rupture from obstructing vesico‐ureteric junction calculus

**DOI:** 10.1002/ccr3.5820

**Published:** 2022-05-09

**Authors:** Emmanuel C. Okpii, Fatima Adamu‐Biu, Kingsley C. Okpii

**Affiliations:** ^1^ North‐West Anglia NHS Foundation Trust Peterborough City Hospital Peterborough UK; ^2^ University Hospitals of Leicester NHS Foundation Trust Glenfield Hospital Leicester UK

**Keywords:** renal calculus, spontaneous renal rupture

## Abstract

Spontaneous or non‐traumatic rupture of the renal tract is an infrequent presentation, and it is most frequently caused by ureteric obstruction. Rupture could occur at any level of the upper urinary tract. However, it is most common at the renal calyces and complications that could arise include; urinoma, and or hematoma collection which could progress to abscess formation and sepsis. We report a 77‐year‐old male patient who attended the emergency department following referral from his general practitioner with a 6‐day history of progressively worsening left sided abdominal pain. Due to his co‐morbidities, presenting blood pressure and age, he was suspected of having an aortic dissection or ruptured abdominal aortic aneurysm and subsequently had a CT (computed tomography) Angiogram. This showed extravasation of contrast from the left kidney with a 12 mm obstructing vesico‐ureteric junction calculus necessitating urgent urology referral and prompt review. He was worked up for a ureteric double J stent insertion, however, the procedure was unsuccessful due to complex multiple urethral strictures. The patient subsequently had a nephrostomy inserted and was planned for optical urethrotomy, rigid cystoscopy, rigid/flexible ureteroscopy, and laser stone fragmentation of left obstructing vesico‐ureteric junction calculus.

## Graphical abstract

Spontaneous renal tract rupture is a rare presentation and commonly results from ureteric obstruction. It should be considered in suspected renal colic and management will, however, depend on the cause and level at which rupture occurs.

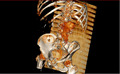

## 1 BACKGROUND

Renal tract rupture is a surgical emergency which results from either trauma or less frequently, spontaneously.^1^ Spontaneous renal tract rupture which is often a sequela of urinary tract obstruction, occurs as a result of increased intraluminal pressure causing upper urinary tract dilatation; hydronephrosis or ureterohydronephrosis. With rupture, urine extravasates forming urinoma which could become infected and subsequently form an abscess.^2^ The evolution of symptoms is dependent on size of rupture; volume of extravasate, and pre‐morbid state of an individual. Small ruptures with contained or occult urinoma could go unnoticed, whereas huge ruptures can rapidly progress to acute abdomen requiring urgent drainage and urinary diversion. Diagnosis of spontaneous renal tract rupture is complicated by a low index of suspicion particularly in patients with several competing diagnoses, however, it should always be considered as a differential in patients presenting with features of renal colic.

## 2 CASE PRESENTATION

A 77‐year‐old male patient was referred by his general practitioner with a 6‐day history of progressively worsening left‐sided abdominal pain. The abdominal pain was of sudden onset and a constant ache scoring 8/10 on the numerical pain scale. His pain radiated to the back with no exacerbating or relieving factors and progressively got worse 3 days prior to presentation.

The patient was an independent man with a WHO (World Health Organization) performance status of zero. With regards to medical history, the patient was a known hypertensive and he had a previous pulmonary embolism. He suffered from chronic back pain from a work‐related injury and also had a suprapubic catheter due to multiple urethral strictures developed post green light prostatectomy. His chronic back pain was a mitigating factor toward his early presentation as he had attributed his chief complaint to an already existing pathology.

General examination revealed an elderly man in no obvious distress. Cardiovascular examination revealed an elevated blood pressure (220/90 mmHg and 224/89 mmHg on the left and right upper limbs, respectively). Abdominal examination revealed a suprapubic catheter in‐situ with no ooze or erythema around the entry port. Abdomen was soft and tender over the suprapubic region. There was no report of costovertebral angle tenderness. Bowel sounds were normal.

## 3 INVESTIGATIONS

**FIGURE 1:**
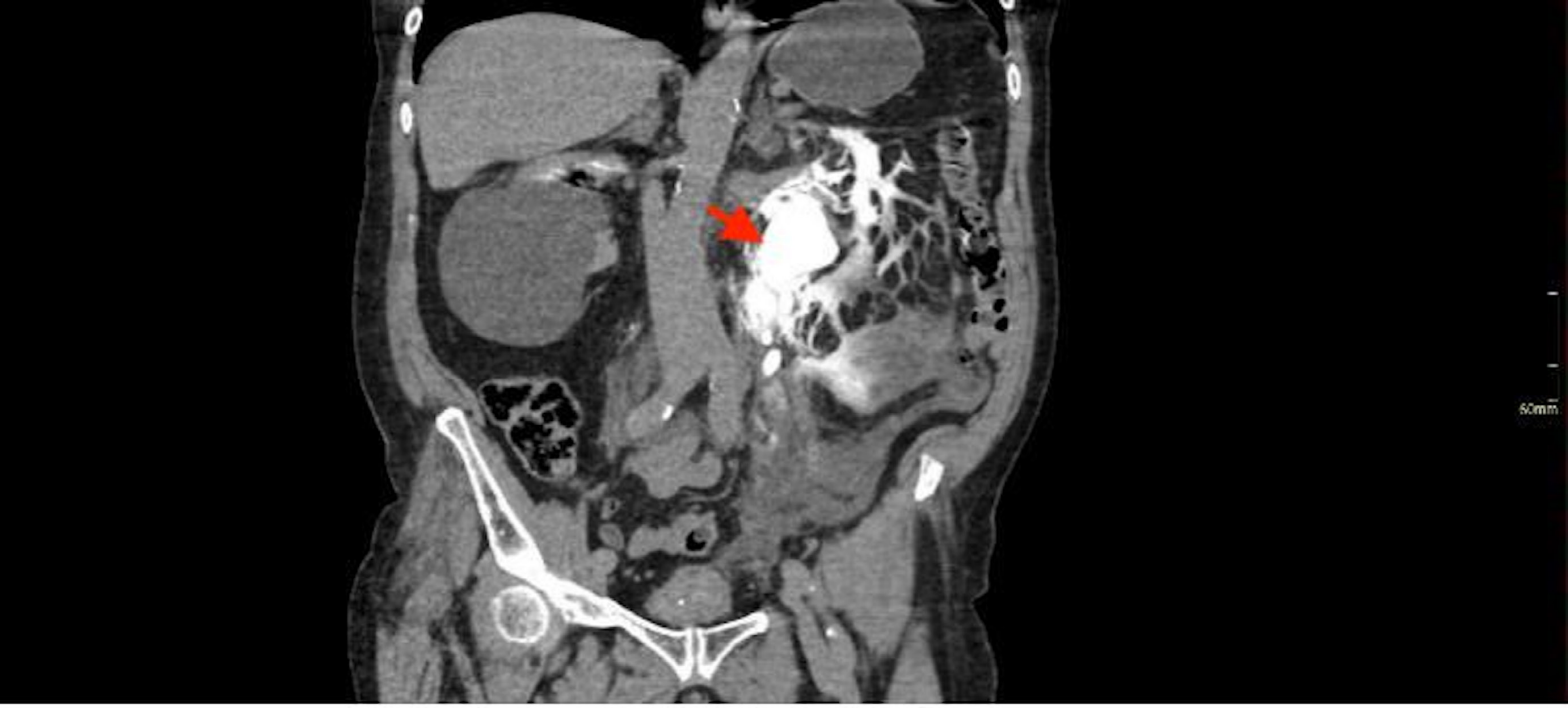
CT Urogram—Coronal section; delayed phase image demonstrating contrast extravasation from the left renal calyx and hold up of contrast in the left excretory system

**FIGURE 2:**
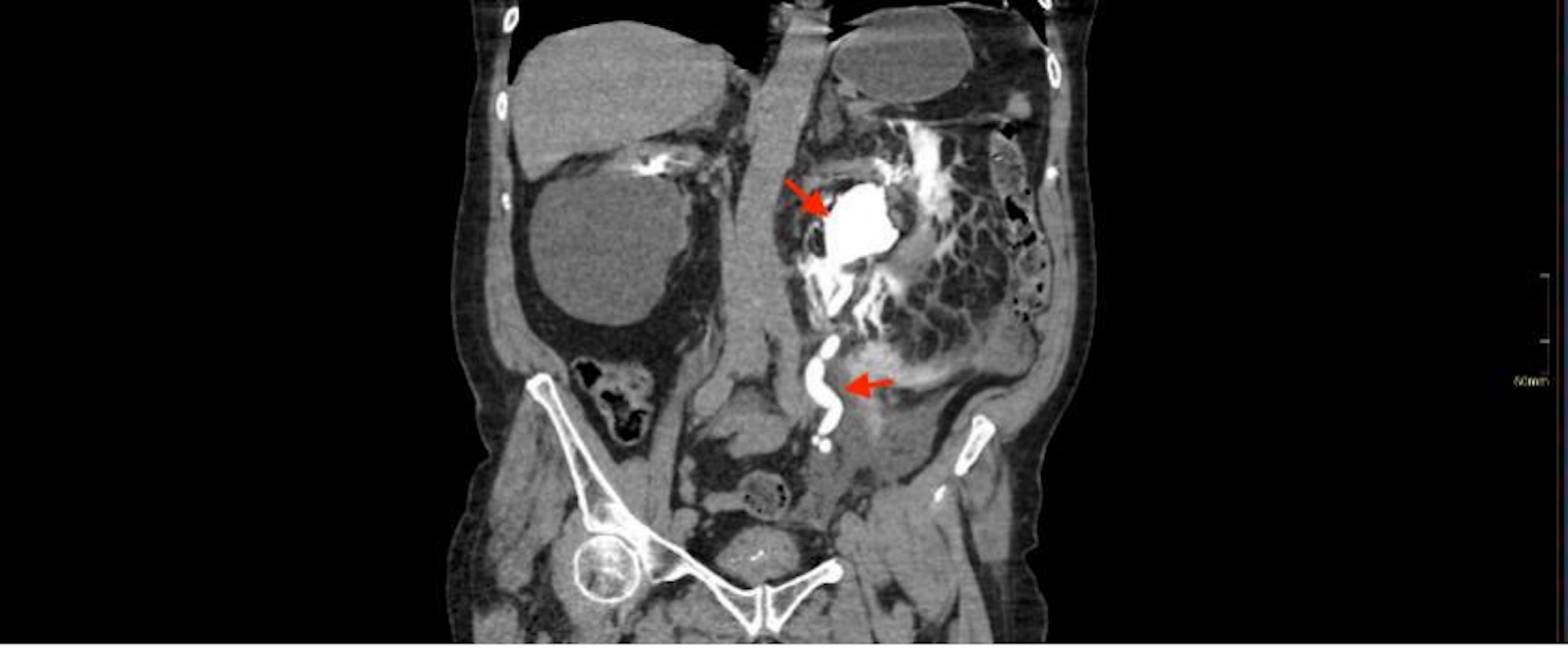
CT Urogram—Coronal section; delayed phase image demonstrating contrast extravasation from the left renal calyx and hold up of contrast in the left excretory system

**FIGURE 3:**
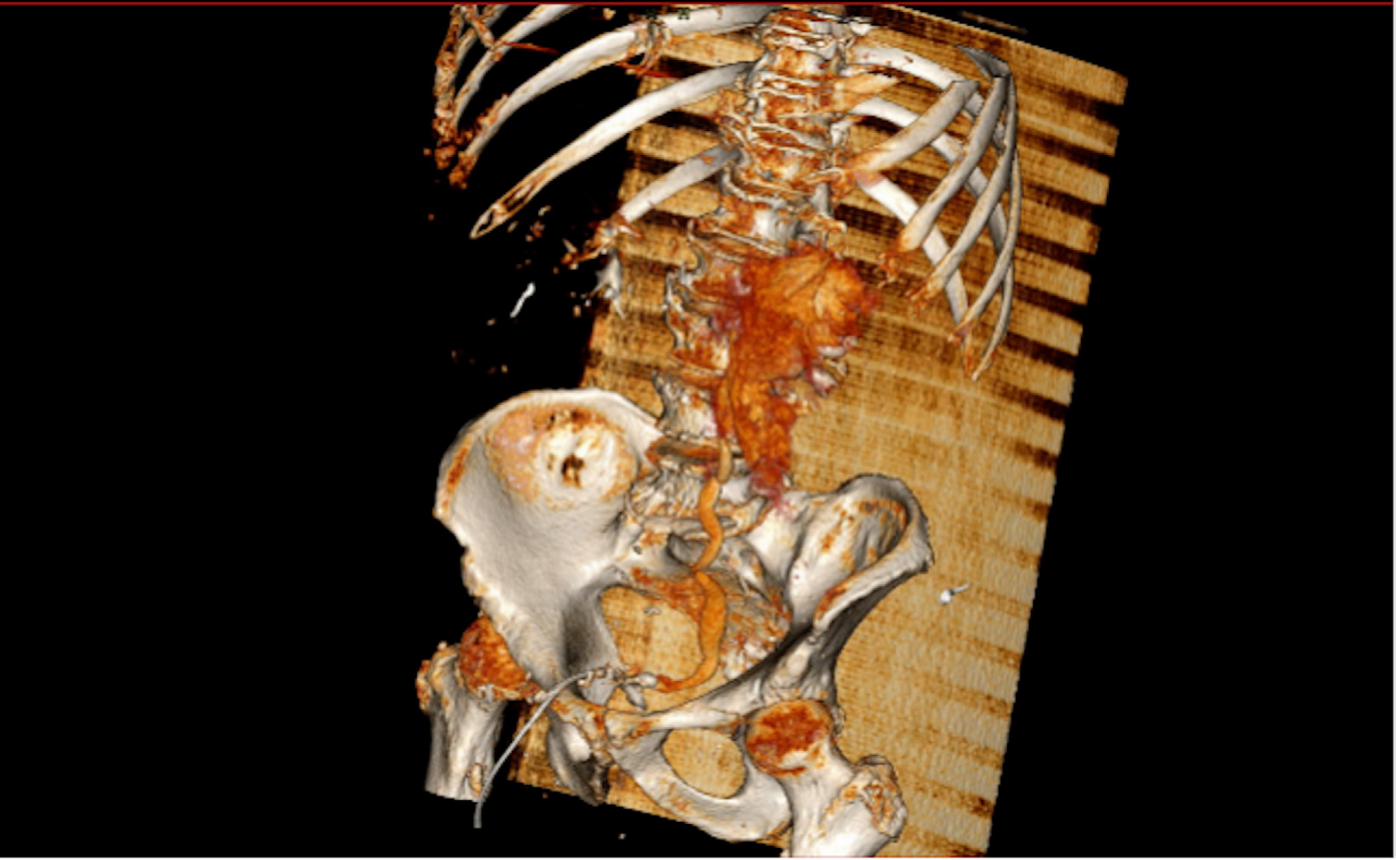
CT Urogram—VR reconstruction; delayed phase image demonstrating contrast extravasation from the left renal calyx and hold up of contrast in the left excretory system due to stone in distal ureter

Full blood count, renal function tests, and serum electrolytes were within normal limits. He was investigated for aortic dissection with a computed tomography (CT) angiogram for suspicion of aortic dissection or ruptured abdominal aortic aneurysm. This revealed an incidental finding of intra‐abdominal free fluid on the left side related to a spontaneous rupture of the left renal calyces due to a 12 mm obstructing left vesico‐ureteric junction calculus (Figures 1, 2 and 3). The patient was promptly referred to the urology team who assessed and discussed investigation findings and options of care with him.

## 4 DIFFERENTIAL DIAGNOSIS

The patient is a known hypertensive with previously documented abdominal aortic diameter reading of 55 mm. Coupled with his age, he was a prime candidate for possible aortic dissection.

## 5 TREATMENT

Upon reviewing him at the emergency department, he was counseled on the findings from computed tomography (CT) angiogram. He was offered a rigid cystoscopy with left ureteric double J stent insertion to de‐obstruct the kidney and to return at a later date for a flexible/rigid ureteroscopy and laser stone fragmentation. He eventually had left nephrostomy on account of difficulty achieving urethral instrumentation due to multiple complex urethral strictures.

There was no immediate post‐operative complication, and patient is awaiting optical urethrotomy, rigid cystoscopy, rigid/flexible ureteroscopy, and laser stone fragmentation of left obstructing vesico‐ureteric junction calculus.

## 6 DISCUSSION

Non‐traumatic renal tract rupture is an infrequently diagnosed urological condition, and outside of its scarce occurrence, a contributing factor to its rarity may be that the diagnostic investigation of choice, a computed tomography urogram, is not first‐line in the management of patients presenting with abdominal and flank pain. Patients presenting with flank pain are investigated with a non‐contrast computed tomography scan or an ultrasound scan if pregnant or children.^3^ Unfortunately, these investigations are not ideally suited to diagnosing a renal tract rupture.

A build‐up of intraluminal pressure within the renal tract is the mechanism that results in spontaneous rupture, and this increased pressure is often the result of ureteric obstruction, commonly by a stone. However, spontaneous rupture can also result in the setting of infections and tumors, which cause increased pressure in the renal pelvis.^4^

Patients with renal tract rupture present with a wide range of symptoms from mild flank pain that goes unreported to more serious symptoms of an acute abdomen resulting from the extravasation of urine. In two reported cases of renal rupture,^2^, ^4^ both patients developed severe flank pain with nausea and vomiting, and both had a history of renal colic in the past.

Computed tomography urogram is the investigation of choice in the diagnosis of urinary collecting system leaks and urinomas. Diagnosis of spontaneous renal tract rupture is complicated by a low index of suspicion particularly in patients with several competing diagnoses. In this case, the patient, a known hypertensive with left sided abdominal pain, was suspected of having a ruptured abdominal aortic aneurysm. Consequently, he had a computed tomography angiogram where an incidental finding of renal tract rupture was made when contrast was seen extravasating into the peritoneal cavity from the lrenal pelvis (Figures 1, 2 and 3).

The management of renal tract rupture is dependent on the level and cause of the rupture. A nephrectomy may be indicated in oncological causes. In other cases, however, immediate urinary de‐obstruction using a ureteric stent or nephrostomy allows the rupture to heal before definitive treatment which usually involves stone removal.^2^, ^4^ Urinoma resulting from urine extravasation may be left to resolve if small, or may require drainage using a catheter.^2^

## 7 OUTCOME

The patient attended for his planned optical urethrotomy, rigid cystoscopy, rigid/flexible ureteroscopy, and laser stone fragmentation 6 weeks after initial presentation. Urethroscopy was, however, unsuccessful due to an obliterated urethral meatus, and thus, cystoscopy was achieved via patient's suprapubic catheter site. Intraoperatively, the stone was not found in the bladder nor the left ureter or its orifice. The patient underwent another CTKUB (computed tomography of kidneys, ureters, and bladder) that showed the stone to be in the prostatic urethra, and he is currently awaiting cystolithotomy or cystolitholapxy.

## AUTHOR CONTRIBUTION

Mr. Emmanuel Chinedu Okpii took the patient's history, wrote and reviewed the manuscript. Ms. Fatima Adamu‐Biu and Dr. Kingsley Chibuikem Okpii wrote and reviewed the manuscript.

## CONFLICT OF INTEREST

The authors declare no conflict of interest.

## ETHICAL APPROVAL

Ethics approval was not required; however, departmental permission was sought prior to publishing.

## CONSENT

The patient's written consent was obtained for the publication of this case report.
